# Supplementary figures and images for: A New NILM System Based on the SFRA Technique and Machine Learning
Source: Sensors (Basel). 2023 May 31;23(11):5226. doi: 10.3390/s23115226 (PMC10256085; doi:10.3390/s23115226)

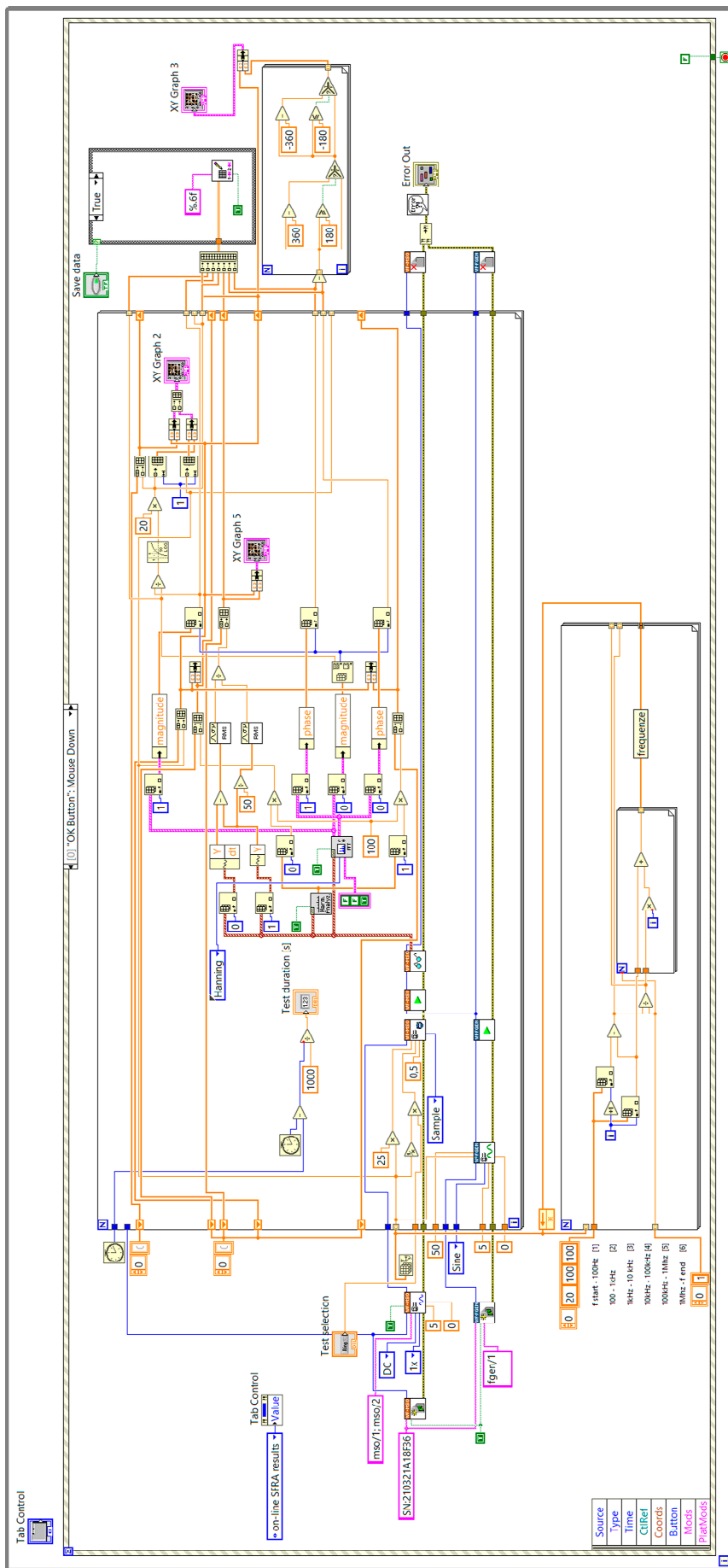

Figure S1. The block diagram of the LabVIEW code.

Supplement: Supplementary file 1 [file sensors-23-05226-s001.zip › sensors-2353748-supplementary.pdf]
